# Thoughts on the construction of public health informatization for community health archives grass roots management system

**DOI:** 10.3389/fpubh.2023.1160478

**Published:** 2023-04-14

**Authors:** Yong Wang, Chaonan Zhu

**Affiliations:** ^1^School of International Finance and Law, East China University of Political Science and Law (ECUPL), Shanghai, China; ^2^School of International Law, East China University of Political Science and Law (ECUPL), Shanghai, China

**Keywords:** public health information, community health records, grassroots management system, information construction, grass roots

## Abstract

With the development of social economy and the continuous improvement of people’s living standards, people expect to receive high-level medical services, and the requirements for medical care are also getting higher and higher. However, there are still objective problems such as rising medical costs, difficulty in seeking medical treatment, uneven distribution of medical resources, low efficiency of medical services, and uneven medical quality. This paper first analyzes the significance of public health informatization construction, focuses on the elements of public health informatization construction, and expounds the status quo of health informatization construction and the existing problems in community health informatization. Then, this paper expounds the construction of public health informatization based on the grass-roots management system of community health records, and discusses the construction of a health information platform centered on the health records of community residents. Afterwards, this paper proposes and studies the functions of the community medical information archives management system from three aspects: the composition of the community medical information archives management system, the problems of system management, and the development requirements of the system, and proposes an algorithm based on a decision tree model to enhance public health informology. Finally, on the basis of experiments and investigations, Internet technology and decision tree model algorithms are introduced into the public health information system construction of the community health archives system to build a new public health information system, and the satisfaction rate can be increased by 23%.

## 1. Introduction

Scientific and orderly management is essential for the normal operation of community health services. In recent years, information management system plays an increasingly essential role in the health field. This is especially true for large and medium-sized hospitals. In these community health service institutions, health record management is gradually becoming computerized. By establishing a regional health information platform and realizing good interaction between health institutions, the overlapping of investment and construction costs in the health sector can be reduced. The construction of an efficient and unified medical resource platform can achieve safe cooperation and profit growth, and effectively enhance the quality of medical services and the service level of medical institutions. This provides people with safe, efficient and affordable public health and basic health services, and effectively solve medical consultation problems.

Grass roots management system is widely used in public health information. Naeem Salman Bin believed that the scale of the crisis and the prevalence of misleading information required scientists, health information professionals and journalists to fulfill their professional responsibilities and help the public identify fake news reports (1). Alsan (2) research found that there was a positive correlation between health protection behaviors and the use of broadcast media as information sources. When used as an information source, unregulated social media might bring health risks, which could be partly but not completely attributed to its role as a propagator of health related conspiracy beliefs. Finney Rutten assessed the progress of health communication and health information technology goals for healthy people in 2020. The goal was to increase the proportion of health information seekers who could easily access health information online (3). Van der Meer (4) conducted an online experiment with samples in order to determine corrective information strategies to raise awareness and trigger actions during the outbreak of infectious diseases. The observed mediation of crisis emotion revealed the mechanism of correcting the influence of information. These findings provided a formula for the study of misinformation to correct the growing spread of misinformation in times of crisis. Sun (5) investigated the current use of the official WeChat account of the Centers for Disease Control and Prevention in public health education, as well as related factors that may affect the effect of information transmission, and carried out a retrospective investigation on the effectiveness of the official account. Jaks Rebecca studied parents’ views on the Internet as a resource for improving health-related knowledge. The information seeking behavior, the type of digital media used, and the reason for use were analyzed descriptively (6). Burr (7) research found that common mental health disorders were on the rise worldwide, which brought pressure to the public health system, and renewed people’s interest in the possible role of digital technology in improving mental health outcomes. The above studies all described the application of public health information, but there are still some deficiencies in community health records.

Many scholars have analyzed and studied community health records. Trevisi (8) studied the impact of community outreach and patient empowerment interventions on the clinical outcomes of diabetes patients. The interventions were aimed at supporting community health development. Abu-Husn (9) reviewed the progress of health information digitization and the proliferation of health system genome research, and provided insights on a new path for the extensive implementation of personalized medicine. The purpose of Petts Rachel A study was to examine the comprehensive behavioral health care experience of patients and providers in health centers. He completed a survey on nursing satisfaction and the degree of integration of the clinic for two patients and providers using a mixed approach design (10). Leis (11) found that in order to improve the influence of antimicrobial management in the community medical environment, the Public Health Bureau had carried out a key activity called the wise use of antibiotics. This campaign was led by antibiotic prescribers themselves, who worked in a community health care environment and were more able to identify specific changes that support more appropriate use of antibiotics. Koleck (12) aimed to synthesize literature on the use of processing or analyzing symptom information recorded in text narratives. Valik (13) aimed to use electronic health record data to develop and verify the fully automatic monitoring system based on in non intensive care units. He proved its practicability by determining the burden of hospital sepsis and the differences between wards. Bellas (14) survey found that community health workers in developing countries often provided door-to-door services in degraded and violent areas. Therefore, he studied the impact of urban violence on performance in underdeveloped areas to understand the challenges of providing care to dangerous communities in developing countries. The above studies have all described the importance of community health records, but there are still some deficiencies in public health information.

In the face of objective problems, health care reform has been accelerated to enhance the level of health management. Unified standards are built to standardize the structure. The construction of a safe and reliable comprehensive medical information platform has become the consensus of the health sector, which takes residents’ health records as the core to build, so as to cover a wide range of network services. This has greatly improved the quality and efficiency of work processing, and is conducive to providing intelligent and professional medical services for the society (15). The government should serve the society and the people to ensure the long-term health development of health care for the benefit of the people and to win the hearts and minds of the government to develop health. To solve these problems, it is necessary to establish a regional information exchange platform centered on residents’ health records to maximize the exchange of medical information and conduct regional information exchange among medical enterprises. Through vertical and horizontal integration, the overall level of health management is improved to achieve a higher level of service. Therefore, the regional health information platform is established to accelerate the growth of medical informatics and provide high-quality medical services. Efficient and intelligent medical services have become an inevitable trend of society (16).

## 2. Importance of public health information construction

### 2.1. Elements of public health information construction

Informatization is the main trend of world economic and social development. Information technology innovation is used to improve work efficiency. The success of medical reform is to build a house on the basis of four beams and eight columns. The first is to enhance the comprehensive development of public health. The second is to further improve the medical security system. The third is to speed up the construction of the medical security system. The fourth is to establish and enhance the drug supply security system. The eight pillars are to establish a unified health management system, an effective mechanism to supervise health institutions, and various health investment systems. The fourth is to establish a scientific and reasonable pricing mechanism for medical care. The fifth is to establish a strict and effective health monitoring system. The sixth is to establish a sustainable innovation mechanism to protect health science and technology talents. The seventh is to construct a comprehensive and universal medical information system. The eighth is to establish and improve the right to health. In short, the four and eight pillars are the public health system, the medical insurance system and drug production. Computerization is the key to everything, and the development of medical information is undoubtedly the most important reform. The introduction of information technology can significantly reduce the cost of various communication and information transmission chains and greatly increase the quality of medical services (17).

### 2.2. Current situation of health information construction

**Figure 1:**
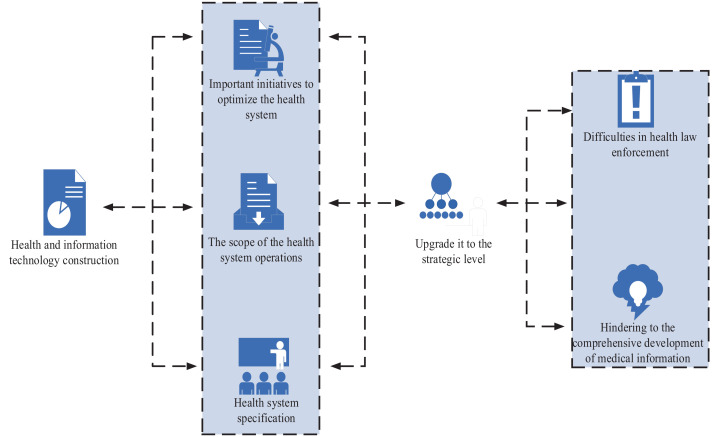
Current situation of health informatization construction.

The active promotion of informatization is a strategic measure for the overall modernization drive, which is an urgent need to realize the scientific outlook on development, and build a well-off society in an all-round way, a harmonious society and an innovative country. Informatization in the medical and health field has become an essential measure to optimize the health system. The health system has upgraded informatization to a strategic level. All departments earnestly practice and constantly improve. Inspired by informatization, the health system has made considerable progress. The health sector is a part of the medical industry, so the information construction needs to start from scratch. The technical requirements in each field are large, and the business scope of the health system is complex and cannot be fully met, such as medical examination items, drug names, etc. Medical terminology and other major standards and codes are much more complex than other information systems (18). Due to the different stages of health development and the objective conditions of some countries in the region, difficulties in coordinating many health law enforcement are also important factors, which hinder the comprehensive development of medical information, as shown in Figure 1. Health information is limited to the traditional governance mechanism. The original drug and health management model has long been aging and has become a closed vertical governance system. Many departments have poor communication and are relatively independent, and information exchange links are weak (19).

### 2.3. Problems in community health informatization

Regional health informatization is regarded as the future development direction of the medical industry, which inevitably encounters difficulties. Because the research on health information creation has been carried out in the community, several different roads and models have been established and explored, and some success has been achieved. However, many problems, such as capital, technology, talents, standards, organization and construction mode, operation mode, legislation, are related to the future growth of medical information in this region. The purpose of construction is to focus on a single project. At present, there is less communication between systems, low cooperative mobility, and the software system has not been fully used reasonably. However, each department maintains its inherent management mode. Information and resources are not shared, and many application concepts are not developed or collaborated. At present, the hospital management, routine immunization, maternal and child health care, emergency command and other information construction methods are independent, not united, and do not understand the purpose of information sharing and information construction. Usually, each department is isolated from the rest of the world. Development needs resources and talents. The growth of medical information involves different disciplines and knowledge, as well as compound knowledge talents in the fields of information and health care.

## 3. Public health information construction based on the community health archives grass-roots management system

### 3.1. Building of a health information platform with community residents’ health records as the core

The main objectives of the health information platform include: urban and rural residents’ information, public health, medical services, medical safety, and drug monitoring. The health information system is established, including important links such as health services. Through the exchange of electronic medical records and electronic medical records, data is exchanged between information systems in different medical and health fields. An identity recognition system with ID card as the main indicator has been established, including the maternal and child health care card, cooperative medical card, medical record information card and other medical and public health related identity recognition means. Community residents are established with health records to provide convenient and high-quality health and medical safety services. On the basis of medical records and medical safety of community residents, a management information system integrating medical service, prevention, health, rehabilitation and education is established. The construction of digital hospitals and digital medical enterprises has been deepened to support hospital management and decision-making, so as to achieve mutual recognition of test results, two-way referral and other medical cooperation (20).

### 3.2. Elements of health information platform construction based on community health archives

The construction of health information platform aims to collect, integrate, store and disseminate health information to ensure the information sharing of various medical institutions. They carry out business cooperation and exchange information with social security departments civil affairs departments, family planning departments and other departments, which provides data support for the cooperation of various health departments, supervision and management support for medical collaboration, and health information services for residents. The Internet is used to provide community residents with information disclosure, information retrieval, health consultation, health improvement, online registration, appointment, remote consultation and other services. The epidemic prevention plan is subject to self-assessment, health management, etc., including community residents’ health card, sharing medical files and cooperation with medical companies. The independent choice of hospitals and doctors is realized to alleviate the difficulties of expensive treatment, including medical resource management, medical service supervision, public health supervision, basic drug distribution management, financial supervision and medical staff performance evaluation.

### 3.3. Functional aspects of health information platform based on community health archives

**Figure 2:**
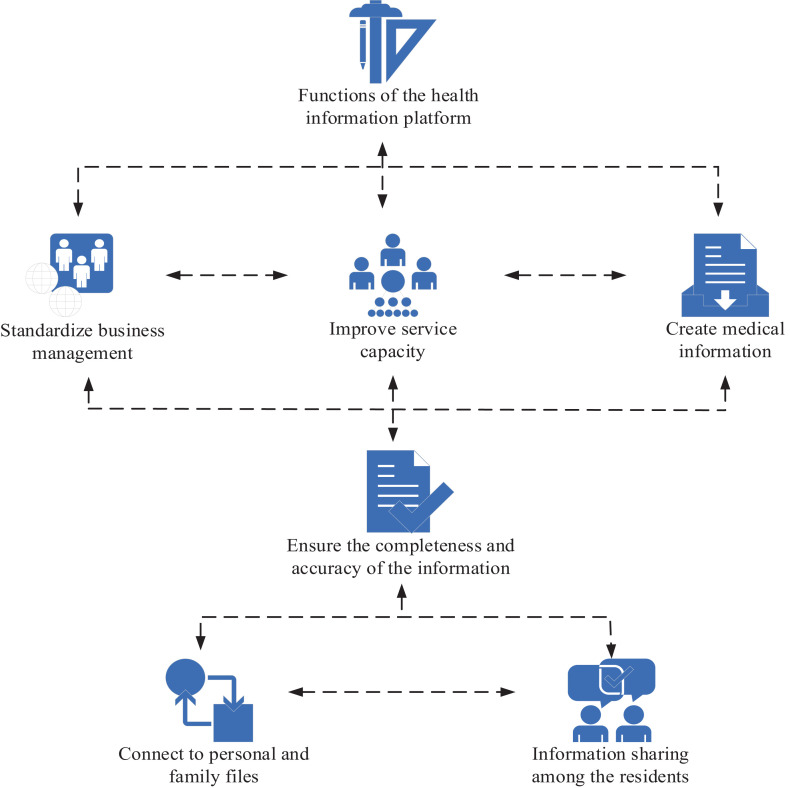
Functions of the health information platform based on community health records.

Service capability is improved by standardizing business management. Standardized community health archives are built to create medical information, such as outpatient service, drug management, hospital medical consultation, chronic disease management, etc. The integrity and accuracy of information are ensured to eliminate errors and accidents and ensure medical safety. Outpatient registration requires examination of forms and other written documents. Patients can consult previous medical records to inform doctors of changes and progress of the disease in a timely manner, so as to make accurate diagnosis. The printed prescriptions and various applications from the doctor’s office can be paid directly, thus reducing the price. This greatly improves the work efficiency and shortens the waiting time and treatment time of patients, thus saving time. When the health card is used, residents can print the test results themselves to reduce cross infection, thus protecting the privacy of patients and eliminating false positives. Grid address embodies the essence of public health management. The grid address is used to connect personal and family files, and connect to the basic medical public health module, eliminate information islands between different systems, so as to ensure information sharing among residents, and achieve network communication. At the same time, patients can record medical history and track chronic diseases to fully reflect the characteristics of public health services, and combine prevention with treatment. The health information of residents is dynamically tracked and collected to timely assess their health status. By strengthening medical quality management, the quality of medical service is guaranteed. The information system should conduct a quality assessment every day to check the work quality of all health departments at any time, so as to find and solve problems in a timely manner, which continuously improves the quality of services, controls the growth of expenditure, and reduces the burden on the public. By controlling the number of days, frequency and single dose of medication, as well as monitoring the average cost, the medical expenses of patients can be controlled to reduce the burden of medical care, and reduce medical expenses. Drug supervision should be improved to achieve dynamic tracking, as shown in Figure 2.

## 4. Functions of community medical information archives management system

### 4.1. Composition of community medical information archives management system

Computer and network technology allows the electronic storage and processing of archival information generated by various medical activities in the community health service system. Data sharing is achieved through information transmission and exchange between community health service centers and urban health service centers, as well as disease monitoring and cause of death analysis (21). The medical management of public health services is fully computerized. End user access tools are developed to identify, report and connect decision support systems to enhance the management of community health services. The intelligent management system includes personal health records, family health records, physical examination management, child immunization, women’s health, children’s health, maternal management, infectious disease early warning system, disease management, personal health records and other modules. Drug use, health education, business accounts, etc. can be managed, which is easy to store and retrieve. Data sharing and real-time reporting are required between health service stations and higher level medical institutions. The connection and correlation between good process planning and results evaluation ensure the reliability, accuracy and objectivity of results, and ensure timely service to the common health information sources of the whole society.

### 4.2. Management and problems of community medical information archives management system

**Figure 3:**
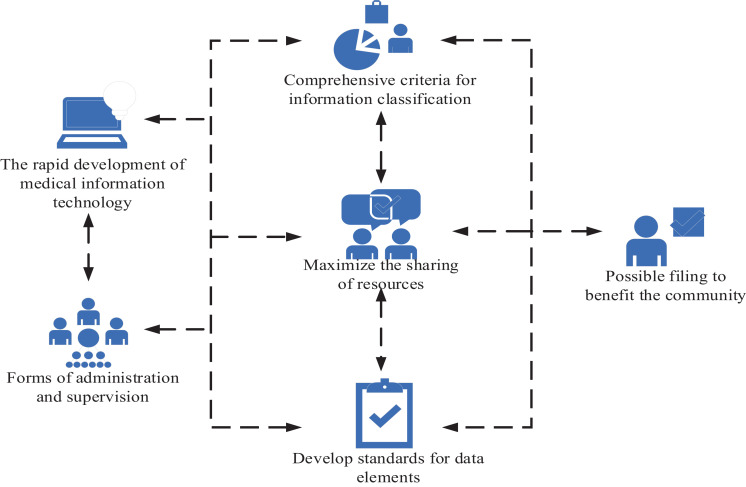
Management and problems of community medical information archives management system.

With the rapid growth of medical information technology, community medical information archives management should be at a higher starting point, preferably in the form of administrative management and supervision, as well as the comprehensive standard of information classification, allowing maximum sharing of resources to prevent geographical differences and low compatibility caused by multi-level development. The development of data element standards and graphic and image transmission standards requires the development of scientific, strict and forward-looking management standards, including organizational management, quality management and technical management. The use of community medical service system resources must comply with the relevant directives and regulations of the Ministry of Health, and ensure the confidentiality of medical records and documents related to the treatment of disabled persons. The system security is strengthened through strict user authority management and password setting to improve modification and other technologies. All regions should be urged to formulate more systematic laws and regulations, regulate the use of relevant information, and take responsibility for illegal use requirements, so that community health information resources can be archived as far as possible to benefit the community, thus ensuring the security of personal privacy and intellectual property rights, as shown in Figure 3.

### 4.3. Development requirements of community medical information archives management system

**Figure 4:**
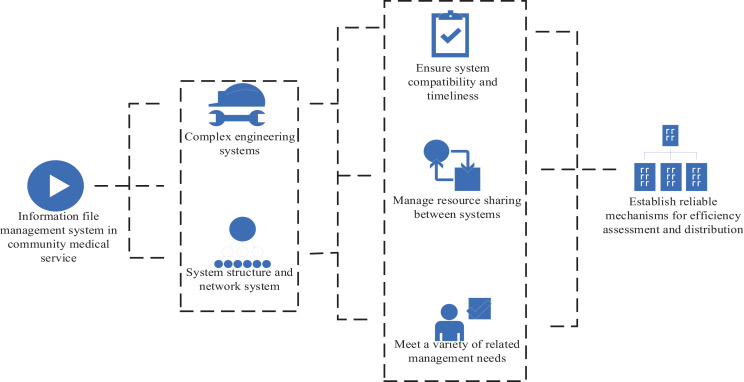
Development requirements of the medical information archives management system in community.

The information archives management system in community medical service is a complex multi department, operational and complex engineering system. Its structure and network system consider the structure and network system of the system from the national or regional perspective. The interface of new methods and new content are maintained to ensure the compatibility and timeliness of the system. The resource sharing between the medical information system and the hospital information management system is improved to meet the needs of reforming and developing the medical service mode through the regional medical information platform. The health data scattered in various institutions is integrated into a logical and complete information base to meet various related management needs. The organic integration of electronic information records, immunization programs, medical information and other professional systems provides comprehensive, sustainable and timely medical service information, as well as data storage and support data markets for management decisions. The health status and risk factors of residents reflected in the community medical service health plan and the health needs analyzed provide a theoretical basis for decision-making and management, and improve the urban health system, thus formulating the community medical service health plan. The online information platform is used to introduce essential drugs, and the detection and use system is established to strengthen drug abuse monitoring. Statistics are compiled to regularly analyse drug abuse in the community. In community institutions, drug abuse is effectively controlled to control the negative trend of drug trafficking. The effectiveness evaluation is integrated to effectively control drug trafficking, so as to establish a reliable efficiency evaluation and distribution mechanism, as shown in Figure 4. The regional health information platform monitors the provision process of public health services, and adjusts the efficiency distribution mechanism according to the total income of community medical service centers and according to the efficiency (22).

## 5. Strengthening of public health information construction based on decision tree model algorithm

The calculation of expected information for a given sample attribute classification is also called information entropy. Information entropy is a more abstract concept in mathematics, which can be understood as the probability of information occurrence. In general, if a certain information has a higher probability of occurrence, it means that it is more widely spread, and it is usually more widely referenced. It is supposed that x is a sample set of public health informatization, and risk assessment y is a different value. It is assumed that x is that the risk level is the number of samples with different values and a certain risk probability. The expected information required for sample classification is:


(1) $$I\left(x_{1},x_{2},x_{3}\ldots ,x_{y}\right)=\overset{y}{\underset{i=1}{\sum }}I\left(x\right)=\overset{y}{\underset{i=1}{\sum }}p\left(i\right)\log\frac{1}{p\left(i\right)}$$

Among them, pi represents the probability of any risk occurrence probability sample.

The average expected information is the estimated information weight of each direct node branch under specific attribute classification conditions to determine the indicators for monitoring public health informatization. In addition, z has multiple different values. Attribute z can be used to divide the sample x of public health informatization construction into several subsets. The average expected information divided into subsets by z is:


(2) $$E\left(x\right)=\overset{y}{\underset{j=1}{\sum }}\frac{z_{j1}+z_{j2}+\ldots +z_{j3}}{z}I\left(z_{j1}+z_{j2}+\ldots +z_{jn}\right)$$

Among them, $\frac{z_{j1}+z_{j2}+\ldots +z_{j3}}{z}$ is the number of public hygiene information samples in the subset divided by the total number of public health emergency samples in z , where:


(3) $$I\left(x_{j1},x_{j2},x_{j3}\ldots ,x_{jy}\right)=\overset{y}{\underset{i=1}{\sum }}p_{ij}\log\left(p_{ji}\right)$$

z The information gain as a branch of the decision tree model is:

## 6. Use of decision tree model algorithm and experimental investigation

At present, with the rapid growth of social modernization, people are paying more attention to their own health, and the management of public health archives in the communities where staff work has become increasingly important. To investigate the current situation of systematization and use of public health information in community health records management, this paper investigated different communities in a city. Community workers were investigated on the current status of public health information systematization and use of health records management. Three communities were selected to conduct a questionnaire survey to investigate the current situation of the use of medical services, drug monitoring, data contact and information sharing in the public health informatization under the management of health records by the staff of these three communities. The three communities surveyed were set as A, B and C, and the number of survey staff was 300. The survey is shown in Table 1.

**Table 1:** The systematic and use of public health information in health records management.

	Medical service	Drug monitoring	Data contact	Information sharing
A	78%	81%	72%	65%
B	63%	73%	68%	81%
C	82%	76%	71%	75%

It can be seen from Table 1 that the current use of public health information systematized medical services managed by health records by community A staff accounted for 78%. The current situation of drug monitoring and use accounted for 81%. The current use of data connection accounted for 72%. Information sharing accounted for 65%. The current use of public health information systematized medical services managed by health records by community B staff accounted for 63%. The current situation of drug monitoring and use accounted for 73%. The current use of data connection accounted for 68%. Information sharing accounted for 81%. The current use of public health information systematized medical services managed by health records by community C staff accounted for 82%. The current use of drug monitoring accounted for 76%. The current use of data connection accounted for 71%. The proportion of information sharing was 75%.

**Figure 5:**
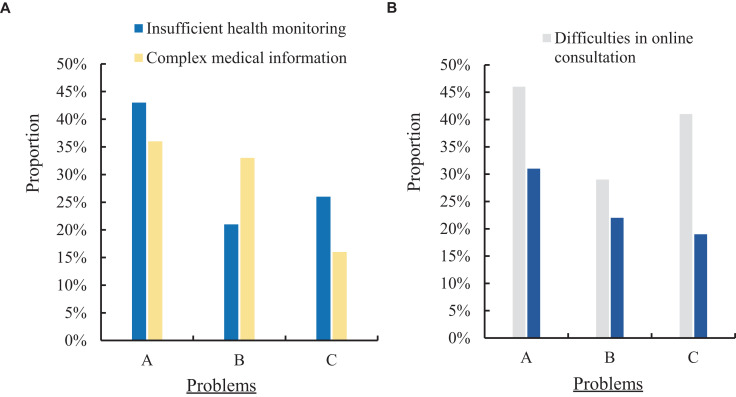
Deficiencies of community health archives management system in the current public health information construction. **(A)** Insufficient health monitoring and complex medical information. **(B)** Difficulties in online consultation and insufficient information resources.

In this paper, the current community health records grass-roots management system of public health information construction status was investigated. The current development status of public health information construction in the grass-roots management system was understood, and residents in three communities were investigated. 150 residents were investigated. In the form of questionnaires, the deficiencies of residents in the current public health information construction of the community health records management system at the grass-roots level were investigated, mainly in the following four aspects: insufficient health monitoring, complex medical information, difficulties in online consultation, and insufficient information resources. The three communities were set as A, B and C respectively, as shown in Figure 5.

Figure 5A shows the proportion of residents in the three communities who have insufficient health monitoring and complex medical information in the current community health records grass-roots management system’s public health information construction. Figure 5B shows the proportion of residents in the three communities who have difficulty in online consultation and lack of information resources in the current community health archives grass-roots management system’s public health information construction. It can be seen from Figure 5A that the residents of the three communities had different situations regarding the insufficient health monitoring and the complex proportion of medical information in the public health information construction of the current community health records grass-roots management system. Among them, 43% of the residents in community A had insufficient health monitoring on the current community health records grass-roots management system in the construction of public health information, and 36% had complex medical information. The proportion of residents in community B who had insufficient health monitoring in the public health information construction of the current community health records grass-roots management system was 21%, and the proportion of medical information complexity was 33%. The proportion of residents in community C who had insufficient health monitoring in the public health information construction of the current community health records grass-roots management system was 26%, and the proportion of medical information complexity was 16%. It can be seen from Figure 5B that the residents of the three communities had different situations regarding the difficulty of online consultation and the insufficient proportion of information resources in the current community health archives grass-roots management system’s public health information construction. Among them, 46% of the residents in community A had difficulties in online consultation on the current community health records grass-roots management system in the establishment of public health information, and 31% had insufficient information resources. The proportion of residents in community B who had difficulty in online consultation in the public health information construction of the current community health records grass-roots management system was 29%, and the information resources were insufficient was 22%. The proportion of residents in community C who had difficulty in online consultation in the public health information construction of the current community health records grass-roots management system was 41%, and the information resources were insufficient was 19%.

**Figure 6:**
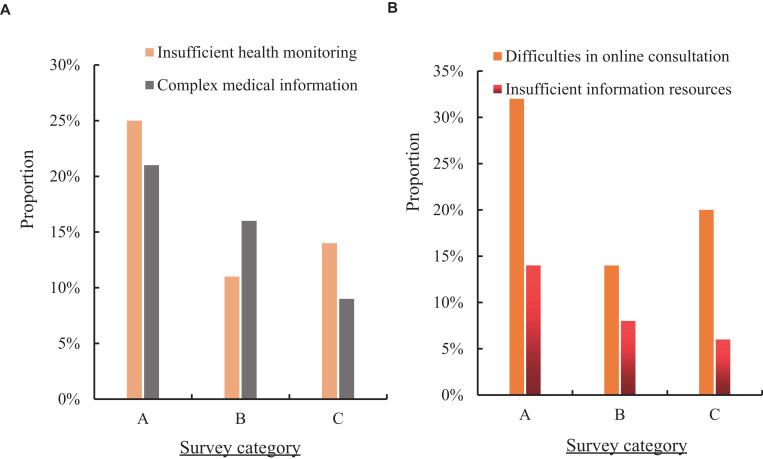
The effect of the new public health information system. **(A)** Insufficient health monitoring and complex medical information. **(B)** Difficulties in online consultation and insufficient information resources.

In order to enhance the current development status of public hygiene information in the community health archives grass-roots management system, this paper introduced Internet technology and decision tree model algorithm into the public health information system construction of the community health archives system to build a new public health information system. To understand the effect of the new public health information system, 150 residents in three communities were investigated. The form of questionnaire was adopted to investigate the proportion of residents in the four aspects of insufficient health monitoring, complex medical information, difficulties in online consultation and insufficient information resources in the new public health information system. The three communities were designated as A, B and C. The specific effect is shown in Figure 6.

Figure 6A shows the proportion of residents in the three communities with insufficient health monitoring and complex medical information in the new public health information system (hereinafter referred to as the new system). Figure 6B shows the proportion of residents in the three communities who have difficulty in online consultation and lack of information resources in the new system. It can be seen from Figure 6A that the proportion of residents in community A who were under monitoring health in the new system was 25%, which was 18% lower than the traditional public health information system (hereinafter referred to as the traditional system). The complexity of medical information accounted for 21%, a decrease of 15% compared with the traditional system. The proportion of residents in community B who had insufficient health monitoring in the new system was 11%, which was 10% lower than that in the traditional system. The complexity of medical information accounted for 16%, down 17% compared with the traditional system. The proportion of residents in community C who lacked health monitoring in the new system was 14%, which was 12% lower than that in the traditional system. The complexity of medical information accounted for 9%, a decrease of 7% compared with the traditional system. It can be seen from Figure 6B that the proportion of residents in community A who had difficulty in online consultation in the new system was 32%, which was 14% lower than that in the traditional system. The information resources were insufficient for 14%, a decrease of 17% compared with the traditional system. The proportion of residents in community B who had difficulty in online consultation in the new system was 14%, which was 15% lower than that in the traditional system. Information resources were less than 8%, down 14% compared with the traditional system. The proportion of residents in community C who had difficulty in online consultation in the new system was 20%, which was 21% lower than that in the traditional system. Information resources were less than 6%, down 13% compared with the traditional system.

**Figure 7:**
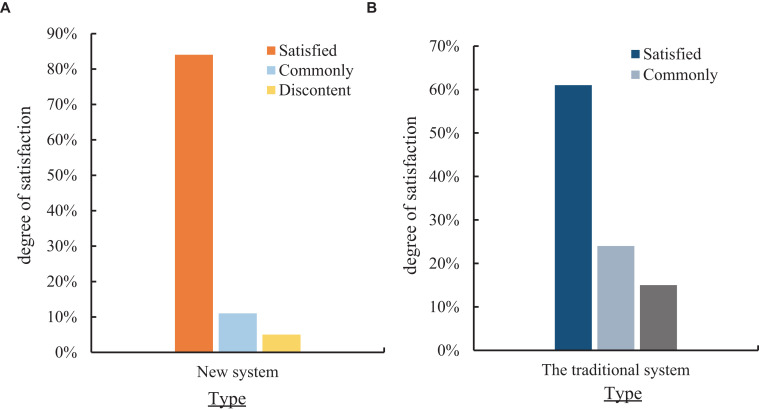
Comparison of residents’ satisfaction with the public health information system of the traditional and new community health records grassroots management system. **(A)** Satisfaction of the public health information system of the new community health records grassroots management system. **(B)** Satisfaction of the public health information system of the traditional community health records grassroots management system.

To understand the different effects of the application of the public health information system and the new public health information system in the new community health records grass-roots management system, this paper investigated the satisfaction of residents in a community with the public health information system of the traditional and new community health records grass-roots management system. 100 residents were surveyed, and their satisfaction was satisfactory, average and dissatisfied, respectively. The specific effect is shown in Figure 7.

Figure 7A shows the satisfaction of residents in a community with the public health information system of the new community health records grass-roots management system. Figure 7B shows the satisfaction of residents in a community with the public health information system of the traditional health records grass-roots management system. As shown in Figure 7A, the satisfaction rate of the new public health information system constructed was 84%. The general rate was 11%, and the dissatisfaction rate was 5%. It can be seen from Figure 7B that the residents were 61% satisfied with the public health information system of the traditional health records grass-roots management system, 24% generally, and 15% dissatisfied. According to experiments and surveys, Internet technology and decision tree model algorithm were introduced into the construction of public health information system of community health archives system to build a new public health information system, which could improve 23% satisfaction.

## 7. Conclusion

In the current social context, information technology has been increasingly applied to the file management, which has become an obvious trend. Community health records are also developing in the direction of information technology. In the current situation, there are still many gaps in the management of community residents’ medical records. First, after summarizing the current situation, improvement measures were proposed. A large number of resources have been used to computerize file management, enabling relevant departments to actively cooperate, thus continuously promoting the development of information management, which has laid an effective foundation for prevention and health education to achieve early prevention. The health and changes of residents were directly recorded in the health records of community residents. The maintenance and use of these records are critical to the health of residents and provide an effective basis for relevant community health work. The reform of the community health system is currently facing serious pressure. The community health record provides an effective framework for work and some effective guidance for people’s prevention and health education. Therefore, it is of great practical significance to divide the current community residents’ health records according to their arrangement and utilization.

## Data availability statement

The original contributions presented in the study are included in the article/Supplementary material, further inquiries can be directed to the corresponding author.

## Author contributions

YW: conceptualization, methodology, software, data curation, writing. CZ: software, visualization, supervision, writing. All authors contributed to the article and approved the submitted version.

## Funding

This work was supported by 2022 excellent doctoral dissertation cultivation project of East China University of political science and Law—Research on universal jurisdiction from the perspective of international law (2022–1-011).

## Conflict of interest

The authors declare that the research was conducted in the absence of any commercial or financial relationships that could be construed as a potential conflict of interest.

## Publisher’s note

All claims expressed in this article are solely those of the authors and do not necessarily represent those of their affiliated organizations, or those of the publisher, the editors and the reviewers. Any product that may be evaluated in this article, or claim that may be made by its manufacturer, is not guaranteed or endorsed by the publisher.
